# Infection control for COVID-19 in hospital examination room

**DOI:** 10.1038/s41598-022-22643-w

**Published:** 2022-10-29

**Authors:** Mamoru Takada, Taichi Fukushima, Sho Ozawa, Syuma Matsubara, Takeshi Suzuki, Ichiro Fukumoto, Toyoyuki Hanazawa, Takeshi Nagashima, Reiko Uruma, Masayuki Otsuka, Gaku Tanaka

**Affiliations:** 1 Safety and Health Organization, Chiba University, 1-33, Yayoi-Cho, Inage-ku, Chiba, Chiba Japan; 2 Department of General Surgery, Chiba University, Graduate School of Medicine, Chiba, Japan; 3 Department of Mechanical Engineering, Graduate School of Engineering, Chiba University, Chiba, Japan; 4 Department of Otorhinolaryngology/Head and Neck Surgery, Chiba University, Graduate School of Medicine, Chiba, Japan

**Keywords:** Environmental social sciences, Health care

## Abstract

Healthcare providers are vulnerable to infection with severe acute respiratory syndrome coronavirus 2 (SARS-CoV-2) because of their close proximity to patients with coronavirus disease 2019. SARS-CoV-2 is mainly transmitted via direct and indirect contact with respiratory droplets, and its airborne transmission has also been identified. However, evidence for environmental factors is scarce, and evidence-based measures to minimize the risk of infection in clinical settings are insufficient. Using computational fluid dynamics, we simulated exhalation of large and small aerosol particles by patients in an otolaryngology examination room, where medical procedures require the removal of a face mask. The effects of coughing were analyzed, as well as those of humidity as a controllable environmental factor and of a suction device as an effective control method. Our results show that a suction device can minimize aerosol exposure of healthcare workers by efficiently removing both large (11.6–98.2%) and small (39.3–99.9%) aerosol particles. However, for coughing patients, the removal efficiency varies inversely with the particle size, and the humidity notably affects the aerosol behavior, indicating the need for countermeasures against smaller aerosols. Overall, these results highlight the potential and limitation of using a suction device to protect against SARS-CoV-2 and future respiratory infections.

## Introduction

The pandemic of coronavirus disease 2019 (COVID-19), caused by severe acute respiratory syndrome coronavirus 2 (SARS-CoV-2), poses a major threat to human health and economies worldwide. For the first time in human history, the spread of this infectious disease has been investigated with relative accuracy, yet it has not been successfully controlled. Healthcare workers are at the forefront of COVID-19 patient care and are constantly at risk of infection. Their safety is therefore paramount to the sustainability of the health care system. Since the beginning of the pandemic, recommended infection control measures for healthcare workers have included the use of personal protective equipment, such as N95 masks, disposable gloves, isolation gowns, face shields and goggles, and the maintenance of careful hand hygiene^1^. Despite the implementation of these measures, many health care workers have been infected. Data from Italy during the first wave of infections show that approximately 20% of Italian healthcare workers were infected, many of whom subsequently died from COVID-19^2^. Current infection control measures are therefore not sufficiently preventive^3^, and collation of evidence for effective and feasible infection control measures remains a critical social issue.

COVID-19 was initially thought to be transmitted by respiratory droplets on surfaces, but much evidence now suggests that it is transmitted through small aerosols similar to other airborne diseases. Transmission of SARS-CoV-2 by asymptomatic or pre-symptomatic individuals (i.e., those without respiratory symptoms such as coughing or sneezing) is thought to account for 33–59%, of cases worldwide, suggesting airborne transmission is the primary route^4^. Direct empirical measurements showed that speaking produces a very large number of aerosol particles but a negligible number of large droplets^5^. Because small aerosols are major carriers of the infectious agent, it is important to consider not only the air streamlines that influence their flow but also the effect of humidity, which can change the aerosol size over time. Transmission of SARS-CoV-2 is more likely to occur indoors than outdoors^6^ and is substantially reduced by indoor ventilation^7^. SARS-CoV-2 has been identified in air filters and building ducts in hospitals with COVID-19 patients; such locations can only be reached by aerosols, which shows the importance of indoor ventilation^8^. To investigate how to efficiently ventilate indoor spaces, it is important to understand air flow as a function of humidity, which has a strong effect on aerosol properties. We have previously investigated and reported infection control methods in medical settings^9^.

Indoor healthcare facilities attract both infected people and patients at high risk of serious illness. Various evidence shows that strict precautions against droplet exposure, such as wearing personal protective equipment and social distancing, are insufficient, and measures to prevent exposure to aerosols are not always well considered^3,10^. Quantification of airborne infection risk using a realistic floor plan and environmental factors is urgently needed to develop feasible effective infection controls and protection strategies for high-risk workers^11^. Few studies have reported on the effectiveness of aerosol removal devices for infection control^12^.

By modeling the airborne transmission of COVID-19 in a hospital outpatient examination room, this study investigated the effects of environmental factors, ventilation, and aerosol suction devices on the spread of SARS-CoV-2 by aerosols. The emergence of highly infectious SARS-CoV-2 subtypes, such as the Omicron strain, indicates that respiratory protective equipment alone may not provide effective protection^3,13^. This finding suggests that optimal aerosol removal methods need to be developed to effectively control COVID-19 transmission in healthcare settings and indoor spaces in general. We therefore examined factors that affect aerosol removal, such as humidity and location of suction devices, and the aerosol exposure of medical personnel to an infected patient’s exhalation and coughing. The generalized risk estimation method used here can be flexibly incorporated into clinical practice to provide feasible infection control.

## Results

### Contributions of suction device to infection control

The 3D model shown in Supplementary Fig. S1 was used to simulate the airborne SARS-CoV-2 infection risk in an examination room designed to perform nasal swabs for viral testing. For the deposition calculations, the aerosol particles were characterized by a different size distributions: small aerosols with a normally distributed average diameter of 10 μm and large aerosols with an average diameter of 80 μm. The sizes of the measured droplets covered four orders of magnitude (0.1–1000 μm). Our study examined the effect of controllable factors such as humidity and the presence and location of an aspirator on the dispersion of exhaled aerosols. In addition, we modeled the dispersion of aerosols expelled during patient coughing.

**Figure 1:**
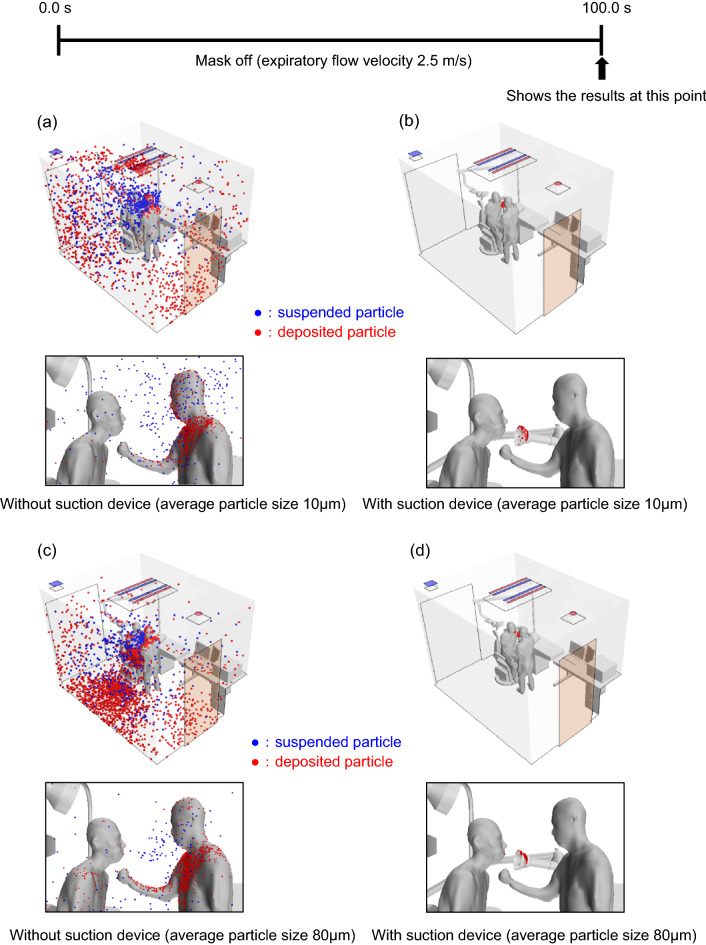
Simulation of suspended (blue) and deposited (red) particle distributions 100 s after the patient begins exhaling at a constant rate of 2.5 m/s (Case X) and assuming no evaporation, in the presence and absence of a suction device positioned as shown in Fig. 2. (**a**) Without suction device; average particle diameter, 10 μm. (**b**) With suction device; average particle diameter, 10 μm. (**c**) Without suction device; average particle diameter, 80 μm. (**d**) With suction device; average particle diameter, 80 μm.

**Figure 2:**
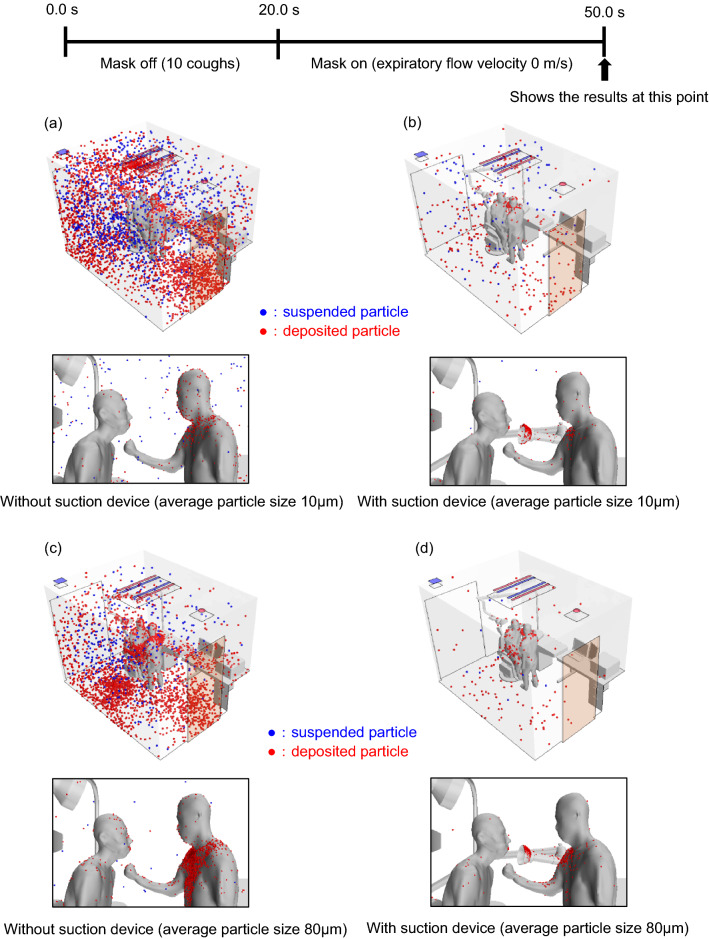
Simulation of suspended (blue) and deposited (red) particle distributions 30 s after the patient completes 10 consecutive coughs modeled by the air velocity profile shown in Fig. 1 (Case Y) and assuming no evaporation, in the presence and absence of a suction device positioned as shown in this figure. (**a**) Without suction device; average particle diameter, 10 μm. (**b**) With suction device; average particle diameter, 10 μm. (**c**) Without suction device; average particle diameter, 80 μm. (**d**) With suction device; average particle diameter, 80 μm.

Figure 1 shows the simulation results assuming a steady exhalation rate of 2.5 m/s (Case X) and no evaporation. When no infection control measures were included in the simulation, small particles (average diameter, 10 μm) were widely suspended and deposited around the entire room (Fig. 1a). Large particles (average diameter, 80 μm) deposited on the floor and the physician more than the smaller particles (Fig. 1c). In comparison, when a suction device (positioned as shown in Fig. 2) was included in the simulation, almost no particles were suspended or deposited around the room, regardless of their size (Fig. 1b,d). Table 1 Case X shows the relative proportions of deposited, suspended, exhaled, and suctioned particles in the room, in the presence and absence of the suction device at a relative humidity of 100% (RH100). Without the suction device, 46.4% of small particles and 79.1% of large particles deposited on the walls, internal structures, and physician, whereas the suction device greatly reduced these numbers to 0.1% and 1.8%, respectively. These results indicate that aerosols exhaled at a rate typical of normal conversation will not be sufficiently eliminated from a medical examination room with standard ventilation and that a suction device can effectively remove them.

**Table 1:** (Case X) The relative proportions of suspended, deposited, exhaled, and suctioned particles in the presence and absence of a suction device, 100 s after the patient begins exhaling at a constant rate of 2.5 m/s and assuming no evaporation (100% relative humidity; RH100).

Case X				
Without suction device				
RH100	All particles	Floating	Adhere	Exhale
Avr. 10 μm	5000	715	2320	1965
	100 (%)	14.3	46.4	39.3
Avr. 80 μm	5000	461	3957	582
	100 (%)	9.2	79.1	11.6

With suction device				
RH100	All particles	Floating	Adhere	Exhale (suction)
Avr. 10 μm	5000	0	7	4993 (4993)
	100 (%)	0.0	0.1	99.9 (99.9)
Avr. 80 μm	5000	0	89	4911 (4911)
	100 (%)	0.0	1.8	98.2 (98.2)

Case Y				
Without suction device				
RH100	All particles	Floating	Adhere	Exhale
Avr. 10 μm	10,000	1111	4087	4802
	100 (%)	11.1	40.9	48.0
Avr. 80 μm	10,000	371	7301	2328
	100 (%)	3.7	73.0	23.3

With suction device				
RH100	All particles	Floating	Adhere	Exhale (suction)
Avr. 10 μm	10,000	102	567	9331 (7712)
	100 (%)	1.0	5.7	93.3 (77.1)
Avr. 80 μm	10,000	9	1248	8743 (7326)
	100 (%)	0.1	12.5	87.4 (73.3)

(Case Y) The relative proportions of suspended, deposited, exhaled, and suctioned particles in the presence and absence of a suction device positioned as shown in Supplementary Fig. S3, 30 s after the patient completes 10 consecutive coughs modeled by the air velocity profile shown in Supplementary Fig. S2 at RH100.

### Effect of cough on aerosol distributions

We next focused on coughing, which produces more aerosol particles than normal exhalation and is thought to be more problematic in clinical practice. Figure 2 shows a simulation of the particle distribution resulting from 10 consecutive coughs (Case Y) modeled by the air velocity profile shown in Supplementary Fig. S2, without considering evaporation. Without the suction device, the distributions of small (Fig. 2a) and large (Fig. 2c) particles were similar to those in Case X, with small particles being widely scattered throughout the room and large particles being deposited more on the floor and physician. However, compared with Case X, particles were scattered over a wider area, and the percentage that adhered to the physician increased. Including the suction device (positioned as shown in Supplementary Fig. S3) in the simulation (Fig. 2b,d) resulted in a larger number of deposited particles compared with Case X (Fig. 1b,d) but a smaller number compared with Case Y without the suction device (cf. Fig. 2a,c). Table 1 Case Y shows the relative proportions of deposited, suspended, exhaled, and suctioned particles in the presence and absence of the suction device at RH100. Without the device, 40.9% of the small particles and 73.0% of the large particles deposited on the walls, internal structures, and physician, whereas the suction device markedly reduced these values to 5.7% and 12.5%, respectively. Although the reduction of deposited particles in Case Y was lower than that observed in Case X (small and large particle numbers reduced by 99.7% and 97.8%, respectively), the number of deposited particles was still reduced by more than 80% in the presence of the suction device. These results indicate that aerosol removal by a suction device can be effective during coughing, despite the reduction in the number of large particles being slightly lower in Case Y (82.9%) than Case X (97.8%).

### Effect of humidity on aerosol distributions

Next, we included the effect of relative humidity in the simulations. The particles did not evaporate in the simulations at RH100, but their size decreased over time at a relative humidity of 75% (RH75). Compared with the previous analysis at RH100, the numbers of small and large suspended, deposited, and excluded particles were lower. The efficiency of the suction system was therefore not markedly affected by humidity. Table 2 shows the relative proportions of suspended, deposited, exhaled, and suctioned particles at RH75 and for a patient exhalation rate of 2.5 m/s (Case X). When the suction device was not present, most of the small particles evaporated immediately after exhalation, and few particles were deposited on the physician or walls. The large particles took longer to evaporate than the small ones, so relatively more large particles were deposited on the physician, resulting in 33.6% of all particles being deposited around the room. When the suction device was present, 97.9% of the large particles were aspirated, and the proportion of deposited particles was 1.3%, which corresponds to a 96.1% reduction compared with the case when the suction device was absent.

**Table 2:** The relative proportions of suspended, deposited, exhaled, and suctioned particles in the presence and absence of a suction device positioned as shown in Fig. 2, 100 s after the patient begins exhaling at a constant rate of 2.5 m/s (Case X) and assuming 75% relative humidity (RH75) and 50% (RH50).

Without suction device					
RH75	All particles	Floating	Adhere	Exhale	Evaporation
10 μm	5000	10	5	0	4985
	100 (%)	0.2	0.1	0.0	99.7
80 μm	5000	149	1681	23	3147
	100 (%)	3.0	33.6	0.5	62.9

With suction device					
RH75	All particles	Floating	Adhere	Exhale (suction)	Evaporation
10 μm	5000	0	0	2602 (2602)	2398
	100 (%)	0.0	0.0	52.0 (52.0)	48.0
80 μm	5000	0	66	4897 (4897)	37
	100 (%)	0.0	1.3	97.9 (97.9)	0.7

Without suction device					
RH50	All particles	Floating	Adhere	Exhale	Evaporation
10 μm	5000	5	0	0	4995
	100 (%)	0.1	0.0	0.0	99.9
80 μm	5000	128	873	0	3999
	100 (%)	2.6	17.5	0.0	79.9

With suction device					
RH50	All particles	Floating	Adhere	Exhale (suction)	Evaporation
10 μm	5000	0	0	1503 (1503)	3497
	100 (%)	0.0	0.0	30.1 (30.1)	69.9
80 μm	5000	0	83	4849 (4849)	68
	100 (%)	0.0	1.7	97.0 (97.0)	1.3

“Evaporation” corresponds to the number of particles that evaporated before they were removed from the room or deposited on the walls.

Table 3 shows the number of small and large particles deposited on the physician in the presence and absence of the suction device at RH75 and RH100. In Case X at RH100, 21.8% of the small particles and 20.9% of the large particles deposited on the physician without the suction device, whereas the inclusion of a suction device reduced these values to zero. At RH75, evaporation reduced the relative number of small and large particles deposited on the physician to zero and 13.5%, respectively. In the Case Y at RH100, 6.4% of the small particles and 33.5% of the large particles adhered to the physician, whereas the inclusion of a suction device markedly decreased both proportions to 1.3%, which corresponds to a reduction of more than 80%.

**Table 3:** Number (a) small and (b) large particles deposited on the physician in the presence and absence of a suction device at 15 cm from the patient’s mouth at RH75 and RH100.

(a) Small particles		
Avr. 10 μm	All particles	Number of particles adhering to the doctor
Case X without suction device (RH100)	5000	1091
	100 (%)	21.8
Case X with suction device (RH100)	5000	0
	100 (%)	0.0
Case X without suction device (RH75)	5000	4
	100 (%)	0.1
Case X with suction device (RH75)	5000	0
	100 (%)	0.0
Case Y without suction device (RH100)	10,000	641
	100 (%)	6.4
Case Y with suction device (RH100)	10,000	128
	100 (%)	1.3

(b) Large particles		
Avr. 80 μm	All particles	Number of particles adhering to the doctor
Case X without suction device (RH100)	5000	1046
	100 (%)	20.9
Case X with suction device (RH100)	5000	0
	100 (%)	0.0
Case X without suction device (RH75)	5000	673
	100 (%)	13.5
Case X with suction device (RH75)	5000	0
	100 (%)	0.0
Case Y without suction device (RH100)	10,000	3349
	100 (%)	33.5
Case Y with suction device (RH100)	10,000	132
	100 (%)	1.3

### Optimal installation position of the suction device

**Figure 3:**
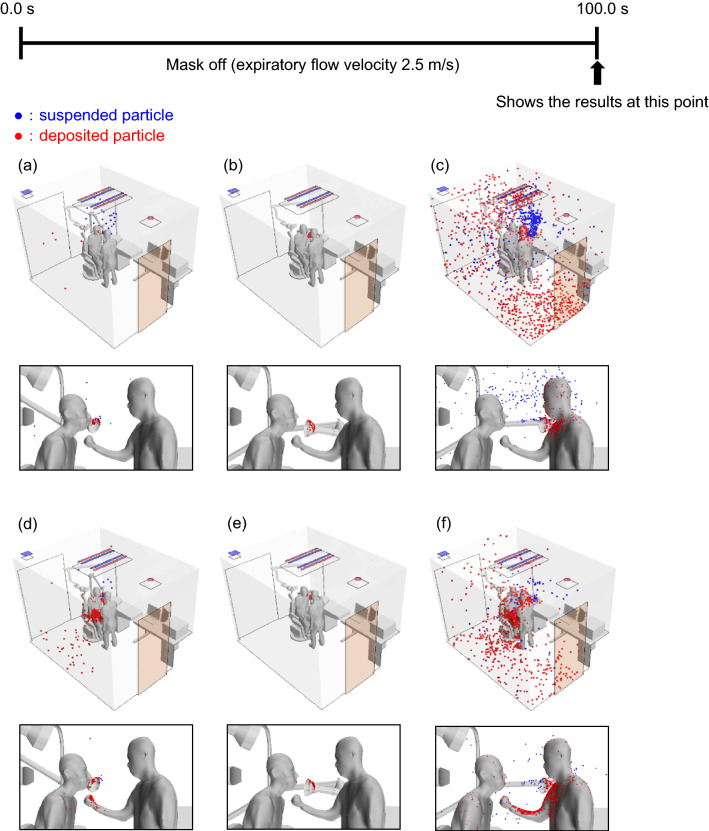
Comparison of simulated distributions of suspended (blue) and deposited (red) particles with the suction device in three different positions shown in this figure, 100 s after the patient begins exhaling at a constant rate of 2.5 m/s (Case X) and assuming no evaporation. (**a**) Suction port placed near the patient’s mouth (small particles). (**b**) Suction port placed approximately 15 cm from the patient’s mouth (small particles). (**c**) Suction port is placed approximately 35 cm from the patient’s mouth (small particles). (**d**) Suction port is placed near the patient’s mouth (large particles). (**e**) Suction port is placed approximately 15 cm from the patient’s mouth (large particles). (**f**) Suction port is placed approximately 35 cm from the patient’s mouth (large particles).

Last, we examined the effect of changing the position of the suction device (Supplementary Fig. S4) on the particle distributions in Case X at RH100. When the suction device was placed near the patient's mouth, most of the smaller particles were eliminated (Fig. 3a) and more large particles were deposited on the patient’s knees and physician’s arms (Fig. 3d) compared with the case where the suction device was placed 15 cm from the patient's mouth (Fig. 3e). When the suction device was placed approximately 35 cm from the patient’s mouth, the numbers of small (Fig. 3c) and large (Fig. 3f) suspended and deposited particles were smaller compared with those observed when the suction device was absent (Fig. 1a–c) but larger than those observed when the suction device was 15 cm from the patient's mouth (Fig. 3b–e). Table 4a shows the relative proportions of suspended, deposited, exhaled, and suctioned particles when the suction device was placed behind the patient's mouth, close to the patient (Supplementary Fig. S4). A large proportion (99.2%) of the small particles was removed by the suction device, similar to the proportion removed when the suction device was placed 15 cm from the patient's mouth (99.9%). However, fewer large particles were removed when the suction device was placed behind (88.9%) rather than 15 cm (98.2%) from the patient's mouth. Table 4b shows the relative proportions of suspended, deposited, exhaled, and suctioned particles when the suction device was placed approximately 35 cm from the patient's mouth. In this instance, only 27.0% of the large particles and 43.4% of the small particles were eliminated by the suction device, much fewer than when the suction port was placed 15 cm from the mouth.

**Table 4:** The relative proportions of suspended, deposited, exhaled, and suctioned particles with a suction device placed (a) beside and (b) 35 cm from the patient’s mouth as shown in Fig. 3, 100 s after the patient begins exhaling at a constant rate of 2.5 m/s (Case X) at RH100.

(a) Placed beside the patient's mouth				
With suction device				
RH100	All particles	Floating	Adhere	Exhale (suction)
Avr. 10 μm	5000	29	10	4961 (4958)
	100 (%)	0.6	0.2	99.2 (99.2)
Avr. 80 μm	5000	13	541	4446 (4443)
	100 (%)	0.3	10.8	88.9 (88.9)

(b) Placed approximately 35 cm from the patient's mouth				
With suction device				
RH100	All particles	Floating	Adhere	Exhale (suction)
Avr. 10 μm	5000	404	1702	2893 (1352)
	100 (%)	8.1	34.0	57.9 (27.0)
Avr. 80 μm	5000	96	2516	2388 (2168)
	100 (%)	1.9	50.3	47.8 (43.4)

### The verification experiments

**Figure 4:**
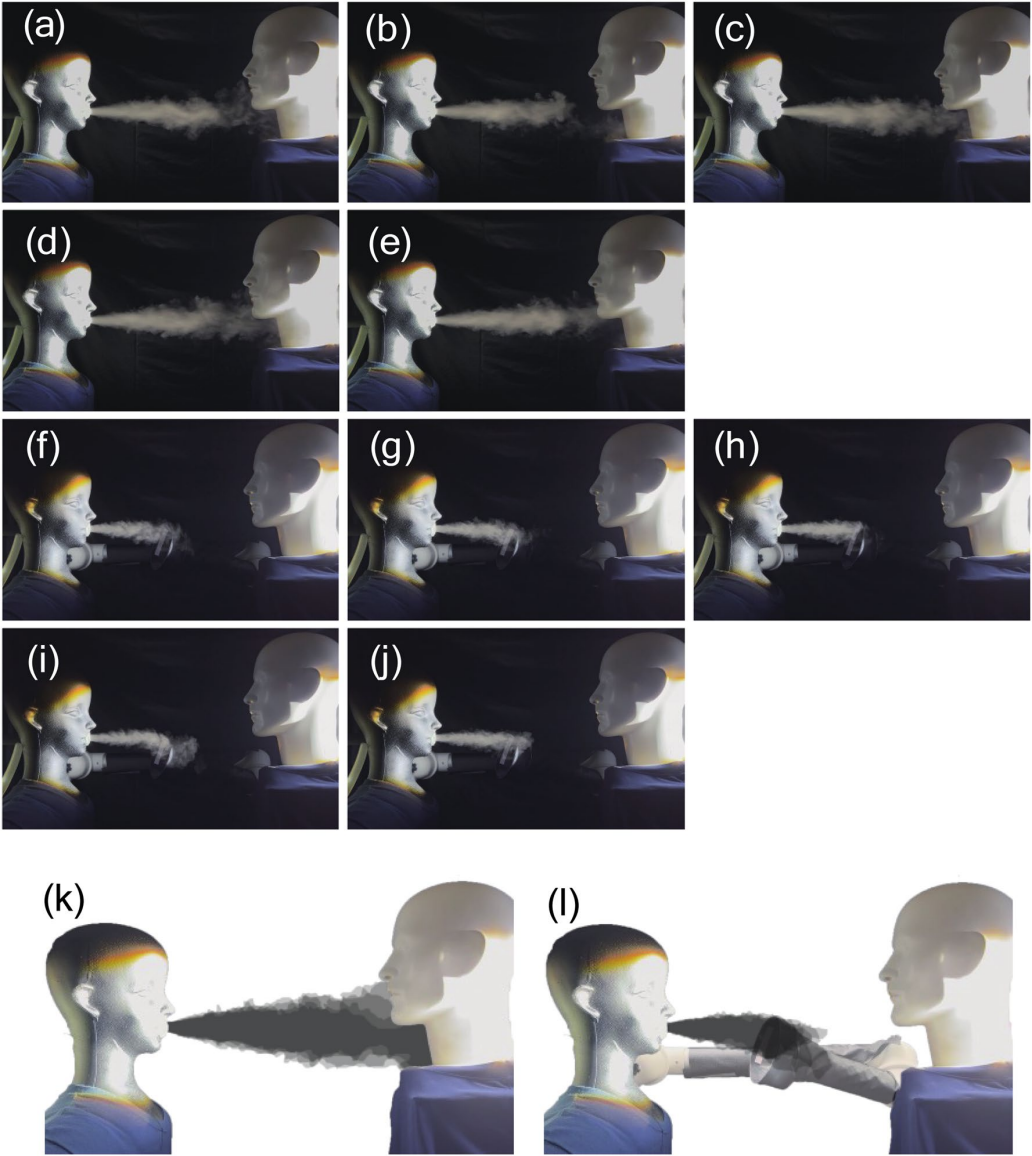
Photographs of the demonstration experiment without suction device (**a**–**e**), and with suction device (**f**–**j**) at 5 s intervals after a sufficient time had elapsed from the start of the mist jetting. Only the mist region of the images was extracted, and the mist region of the five pictures was layered without suction device (**k**) and with suction device (**l**).

To validate the simulation study, a mist aerosol validation experiment was conducted at the Otorhinolaryngology Department of Chiba University Hospital (Fig. 4). The experiment was reproduced using two mannequins and a suction machine with the same positioning and suction machine angle as the simulation-optimized results in Fig. 3e. The room temperature was 25 °C and the relative humidity was 74%. Mist was emitted at a velocity of 2.5 m/s from a mannequin simulating a patient to a mannequin simulating a doctor, and the mist was observed without and with a suction machine, respectively. Without a suction device, the mist emitted from the patient was dispersed in front of the medical personnel. On the other hand, when a suction device was used, the mist decreased between the suction device and the examinee. The results of this experiment are in good agreement with our computational model.

## Discussion

The results of this theoretical study clearly show that aerosols emitted from a patient's mouth or nose in an examination room, where masks must be removed, are greatly affected by the airflow in the room, and the risk of their deposition on the physician and elsewhere in the room cannot be neglected (Fig. 1). This finding is consistent with our previous report^9^. Furthermore, the movement of aerosols was modeled under the assumption that a patient coughs, and it was found that the number of aerosols deposited on the physician was comparable with that resulting from normal patient expiration, although small aerosols diffused widely throughout the room (Fig. 2).

The effect of humidity was also examined in this study. Although humidity is an easily controllable environmental factor in the clinic, few previous studies have examined the impact of humidity on SARS-CoV-2 infection. Humidity affects the mucous membranes of the upper respiratory tract in both infected and uninfected individuals, but small aerosols might have a greater impact. Our simulations show that a the diameter of large aerosol particles is reduced within seconds at RH75, a humidity that is higher than that of New York City, USA, all year round^14^. This suggests that more attention may need to be paid to controlling smaller aerosols to limit airborne transmission of SARS-CoV-2.

Compared with RH100, the number of suspended, deposited, and excluded particles were lower at RH75 because most particles evaporated within approximately 7 s, indicating that humidity has a marked effect on particle size.

The humidity in the examination room at our facility was relatively higher (74%; the weather was cloudy). Japan is a relatively humid environment, and lower humidity simulations should be considered globally. In our CFD model, relative humidity is imposed on the air conditions of the entire room. First, simulations were conducted at 100% relative humidity (RH100) to evaluate the highest aerosol exposure risk, since aerosols theoretically do not evaporate in RH100. The simulations were performed at RH75 because the actual humidity in the otorhinolaryngology examination room at Chiba University Hospital is approximately 75% (74%). The simulation was performed again at RH50 (Table 2) as well. The results show that aerosols evaporate faster at RH50 than at RH100 and RH75, indicating a lower risk of aerosol exposure at lower humidity levels.

In the present simulation, the local temperature was not given because it was not considered to have a significant effect on the conclusions. However, in order to clarify the influence, we performed the analysis considering the actual local temperature. The results with actual environmental considerations (mouth temperature, droplet temperature, body temperature, and mouth humidity) of the analysis are compared those without at room humidity of RH50, room temperature of 25 °C, and 100 s. Based on a previous study, the set values were determined to 33 °C for the mouth temperature and 31 °C for the body temperature^15,16^. The droplet temperature was set to the same temperature as that of the mouth. The humidity in the mouth was set to RH70. The results of the simulation are shown in Supplementary Table S.

According to the results, for 10 μm particles, the effect was 0.1%, even after detailed consideration of the actual environment. For 80 μm particles, the effect was at most in the 1–2% range. Hence, these results suggest that the effect of local temperature is small in this study.

For particles with extremely small mass, it is possible to simulate their behavior by solving the coupled flow and particle equations. It is also possible to estimate their behavior without solving the particle equations by instead solving the equations of flow and integrating the velocity distribution of air from an appropriate initial position, such as the mouth. In our simulations, we considered random particle behavior due to macroscopic turbulence rather than microscopic Brownian motion.

In terms of airborne infection by aerosols, the number of droplets aspirated into the body from a macroscopic point of view is likely more relevant than the microscopic particle behavior (collision and deposition). Therefore, in this study, particles with negligible mass were substituted by streamlines.

Several physical barriers have been designed to protect healthcare workers during aerosol-generating activities, but these devices prevent direct contact with larger droplets and have not proven effective against smaller aerosols, which are considered more important^17^.

A steady flow of 2.5 m/s was used to model normal patient exhalation. Although the full respiratory waveform could also be used, this may not affect our conclusions regarding infection control because it would only reduce the aerosol released into the room.

To develop more efficient and feasible countermeasures against SARS-CoV-2, we also simulated the efficiency of aerosol removal using a suction device. The results showed that aerosol dispersion during normal expiration (Fig. 1) and coughing (Fig. 2) can be more effectively removed by a suction device than by relying on the maximum efficiency of airflow in the room (data not shown).

In clinical settings, respiratory events such as coughing or sneezing are likely to occur during otolaryngology consultations and nasal swab procedures for SARS-CoV-2 testing^18,19^. Although common sense-based general measures have been recommended for such high-risk practice settings, no evaluation of suction devices has been reported to date^20^. Our analysis showed that particle removal efficiency of the suction device was lower in response to coughing compared with normal exhalation. Therefore, more effective infection control measures might be needed against more infectious mutants and diseases that result in more frequent coughing and sneezing.

It is important that a suction device used in the examination room is extensible and does not interfere with the examination.　The size and extensibility of the device in this study were designed to be compatible with various examination and operating rooms in real clinical environments. The efficiency of aerosol removal was investigated as a function of the device location. The results suggest that it is important to place the vacuum inlet of the suction device in a ‘direction’ facing the patien’s mouth rather than a ‘distance’ in which it is closer to the patient exhaling pathogenic aerosols. The simulated diameter and suction speed of the device were 6 cm and 1.2 m^3^/min, respectively, and although it is not necessary to use a higher suction speed, we did not examine speeds lower than this.

A limitation of our model is that the number of aerosols that act as vectors of the virus is not known, so the risk can only be estimated from their distribution, which cannot be evaluated quantitatively. In addition, the respiration of the physician is not considered because it is difficult to model the exhalation and inhalation of a physician wearing a mask. In addition, only three positions of the suction device were investigated, and these may not correspond to the optimal placement.

The simulation results were reproduced in the verification experiment (Fig. 4). As for the optimization of the position of the suction machine, by placing the suction machine at 15 cm from the patient model’s mouth, with the suction device facing the patient model side, no visible mist could be seen around the doctor model (Fig. 4). This position of the suction machine was confirmed by the otolaryngologist (TS) not to interfere with the examination.

In conclusion, we have discussed the inadequacy of infection control measures, such as opening windows and doors, against aerosols in the examination room and the increased risk to health care providers from coughing. We have also shown that humidity notably changes the behavior of aerosols, indicating that suction devices can increase safety in our limited clinical applications. With further refinement of the model parameters, our approach may guide the design of devices that can more effectively limit the spread of respiratory diseases including COVID-19.

## Materials and methods

### Model

Using three-dimensional (3D) computer-aided design software (Fusion 360, Autodesk), we constructed a virtual model of an otolaryngology consultation room in the outpatient clinic at Chiba University Hospital, Japan (Supplementary Fig. S1a). The geometries of the inlets and outlets of the air-conditioning unit and their associated wind directions (red and blue areas in Supplementary Fig. S1) were measured and used in design drawings to simulate aerosol dispersion in the clinic. The airflow rate was determined using data in the architectural drawings. Surface renderings of the patient and examiner (Supplementary Fig. S1b,c) were obtained by scanning a person 175 cm tall using a hand-held 3D scanner (3D Scanner 2.0, XYZ Printing).

### Computational fluid dynamics simulations

To simulate airflow in the model otolaryngology consultation room, the 3D unsteady Reynolds-averaged Navier–Stokes equations were solved with the k–ω shear stress transport turbulence model using the STAR-CCM + software (Ver. 16.02; Siemens Digital Industries) with a second-order segregated flow solver based on the semi-implicit method for pressure-linked equations. The working fluid was air, an ideal gas, at normal temperature and pressure (25 °C, 1 atm), and two levels of relative humidity (100% and 75%) were analyzed. Computational hybrid meshes of the otolaryngology consultation room were generated by combining interior polyhedral elements with a five-layered prism mesh adjacent to the wall. The minimum prism height was 30 μm, and the total number of meshes was approximately 6 million.

As a boundary condition, a flow velocity of 1.58 m/s (0.023 m^3^/s) was imposed at the inlet above “Door A”, 0.52 m/s (0.023 m^3^/s) was imposed at the outlet in the ceiling, and 1.6 m/s (0.063 m^3^/s) was imposed at the outlet and inlet of the air conditioner (Supplementary Fig. S1a). Following the conditions present during a medical examination, Door A was closed and a constant atmospheric pressure was imposed at the open Door B. A no-slip condition was applied to the other walls.

The flow perpendicular to the opening of the human mouth (Supplementary Fig. S1d) was analyzed in two cases: (i) a constant expiratory rate of 2.5 m/s (0.00075 m^3^/s)^[X]^ (Case X) and (ii) 10 consecutive coughs using flow waveforms (Supplementary Fig. S2) obtained from previous studies^[Y]^ (Case Y)^21^. Case X models an examination using an otoscope, during which the patient’s mask must be removed, and assumes an expiration rate typical of normal conversation^22^. Case Y models coughing, a major symptom of COVID-19, during examinations requiring the removal of the patient’s mask. Cases X and Y were analyzed using a time resolution of 0.1 and 0.02 s, respectively.

From the initial conditions (pressure and flow velocity 50 s after the room air conditioning was turned on), the airflow and particle tracking was simulated for 100 s in Case X and 50 s in Case Y, respectively. The discrete phase model in the STAR-CCM + software was used to calculate particle tracks by integrating the particle velocity obtained from the equations of particle motion in the Lagrange formulation. Two-way coupling was assumed between the flow and particle equations. The particles were represented using a Rosin–Rammler distribution with a minimum diameter of 0.1 μm, an average diameter of either 10 or 80 μm, and a maximum diameter of 1000 μm^23–25^. The particle density was assumed to be equivalent to that of water and evaporate in a quasi-steady manner in response to humidity. The Spalding evaporation model based on the Sherwood and Nusselt numbers was selected to consider mass and heat transfer between the droplet surface and the surrounding air. The droplet was assumed to be internally homogeneous. The Sherwood and Nusselt numbers were calculated using the turbulence Reynolds number, which was calculated using the turbulence length and time scales in the turbulence model. In Case X, particles were emitted from the mouth at one of five random points that changed position at each time step (0.1 s). In Case Y, one particle was emitted from the mouth when the flow rate was positive from one of 40 random points that changed position at each time step (0.02 s), and 1000 particles were emitted per cough.

In this study, we performed simulations with and without a suction device positioned near the patient. The device was assumed to be of a size compatible with the room, and its 3D shape was built using computer-aided design. Air and aerosols were aspirated at a rate of 6.71 m/s (0.02 m^3^/s) into a suction port at the end of a flexible arm (Supplementary Fig. S3) and expelled at a rate of 5.66 m/s (0.02 m^3^/s) from an exhaust port at the rear of the device. This speed was set within the range of noise that does not interfere with medical treatment based on the results of tests at actual sites. The aspirated aerosol was assumed to be removed by a filter in the device. As shown in Supplementary Fig. S3, the suction port was positioned to one side of the patient, being 10 cm forward from their face and 15 cm from their mouth. To investigate the effect of the position of the suction device on aerosol removal, simulations were also conducted assuming the suction device was placed near the patient's mouth and 35 cm from their mouth (Supplementary Fig. S4).

### Validation experiments

Validation experiment was conducted using a fine water mist and an LED light source to visualize particle dispersion in an actual otorhinolaryngology laboratory at Chiba University Hospital. An ultrasonic humidifier that emits aerosols with sizes ranging from several to dozens of micrometers was connected to a mannequin's breathing apparatus. The exhalation velocity was adjusted to be the same as in the CFD simulation (2.5 m/s). The room temperature and humidity were 25 °C and 74%. Mist images were recorded with a digital camera (iPhone 13 Pro, Apple). Five mist images were recorded at 5-s intervals after enough time had elapsed from the start of mist emission (Fig. 4). Using the area expansion tool of image editing software (Photoshop, Adobe), only the mist area of the image was extracted, and the mist areas of the five photographs were layered.

## Supplementary Information

Supplementary Information.

## Data Availability

The data that support the findings of this study are available from the corresponding author, M.T., upon reasonable request.
