# Supplementary material for: Infection control for COVID-19 in hospital examination room
Source: Sci Rep. 2022 Oct 29;12:18230. doi: 10.1038/s41598-022-22643-w (PMC9617229; doi:10.1038/s41598-022-22643-w)
Supplement: Supplementary file 1 — Supplementary Information. [file 41598_2022_22643_MOESM1_ESM.docx]

Supplementary Information for

Infection control for COVID-19 in hospital examination room

Mamoru Takada^1,2*^, Taichi Fukushima^3^, Sho Ozawa^3^, Syuma Matsubara^3^, Takeshi Suzuki^4^, Ichiro Fukumoto^4^, Toyoyuki Hanazawa^4^, Takeshi Nagashima^2^, Reiko Uruma^1^, Masayuki Otsuka^2^, Gaku Tanaka^3^

1, Safety and Health Organization, Chiba University, 1-33, Yayoi-cho, Inage-ku, Chiba-city, Chiba, Japan

2, Department of General Surgery, Chiba University, Graduate School of Medicine, Chiba, Japan.

3, Department of Mechanical Engineering, Graduate School of Engineering, Chiba University, Chiba, Japan.

4, Department of Otorhinolaryngology/Head and Neck Surgery, Chiba University, Graduate School of Medicine, Chiba, Japan.

*Corresponding: mamoru@chiba-u.jp

Table of contents

Supplementary Figure S1. Model of an otolaryngology consultation room in the outpatient clinic at Chiba University Hospital, Japan. Shown are (a) the geometry of the clinic, surface renderings of the (b) patient and (c) physician, and (d) the dimensions of the patient’s mouth.

Supplementary Figure S2. Model particle velocity profiles perpendicular to the opening of the patient’s mouth. Simulations assumed either (a) a single cough or (b) 10 consecutive coughs.

Supplementary Figure S3. Layout of the suction device.

Supplementary Figure S4. Three suction port arrangements modeled in this study.

Supplementary Table S. Simulations considering the actual local temperature.

Setting conditions details: Mouth temperature 33°C, Mouth humidity RH 70, Droplet temperature 33°C, Inspectors/inspectors 31°C, Room temperature 25°C, Room humidity RH50

Figure S1. Model of an otolaryngology consultation room in the outpatient clinic at Chiba University Hospital, Japan.

Figure S2. Model particle velocity profiles perpendicular to the opening of the patient’s mouth. Simulations assumed either (a) a single cough or (b) 10 consecutive coughs.


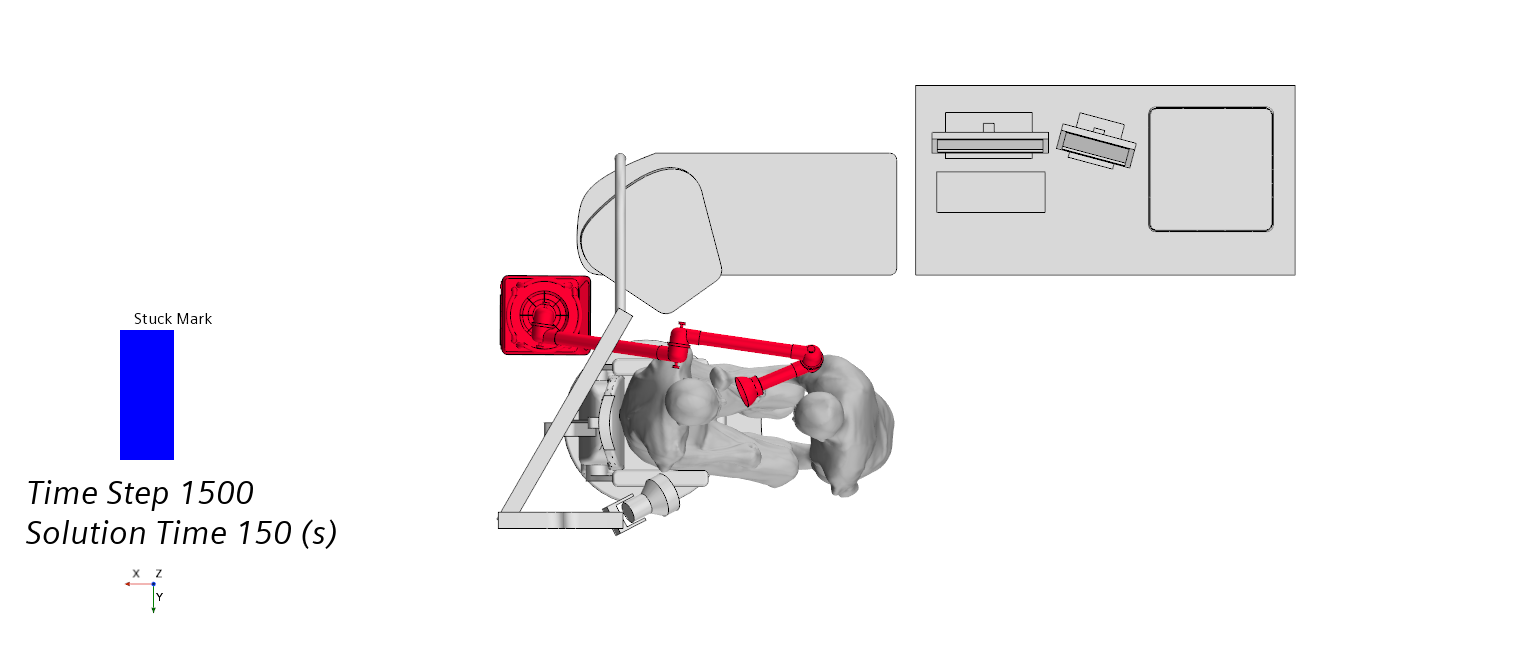

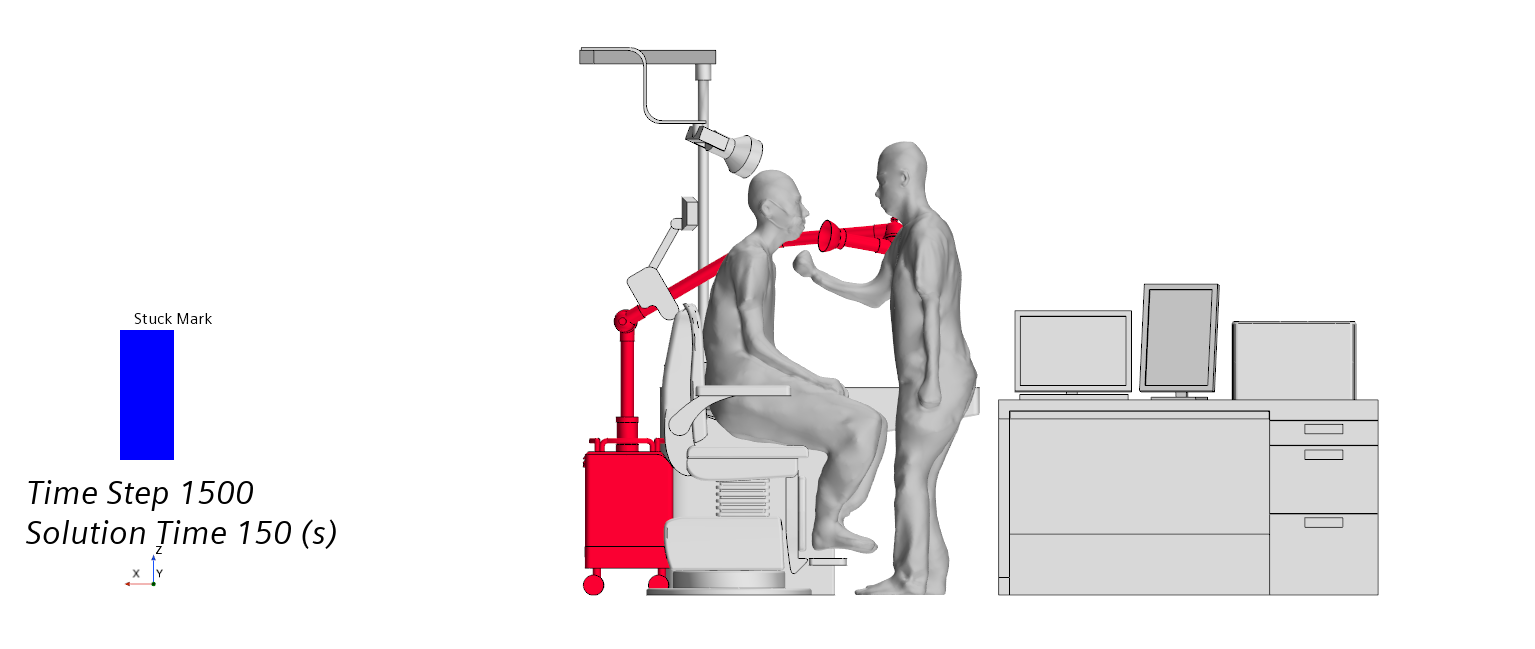

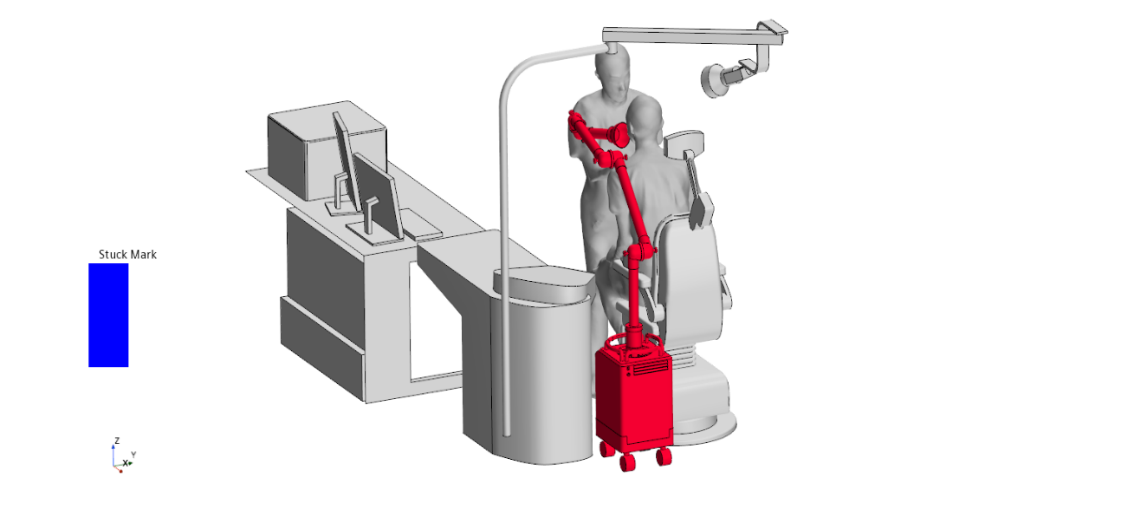


Figure S3. Layout of the suction device.


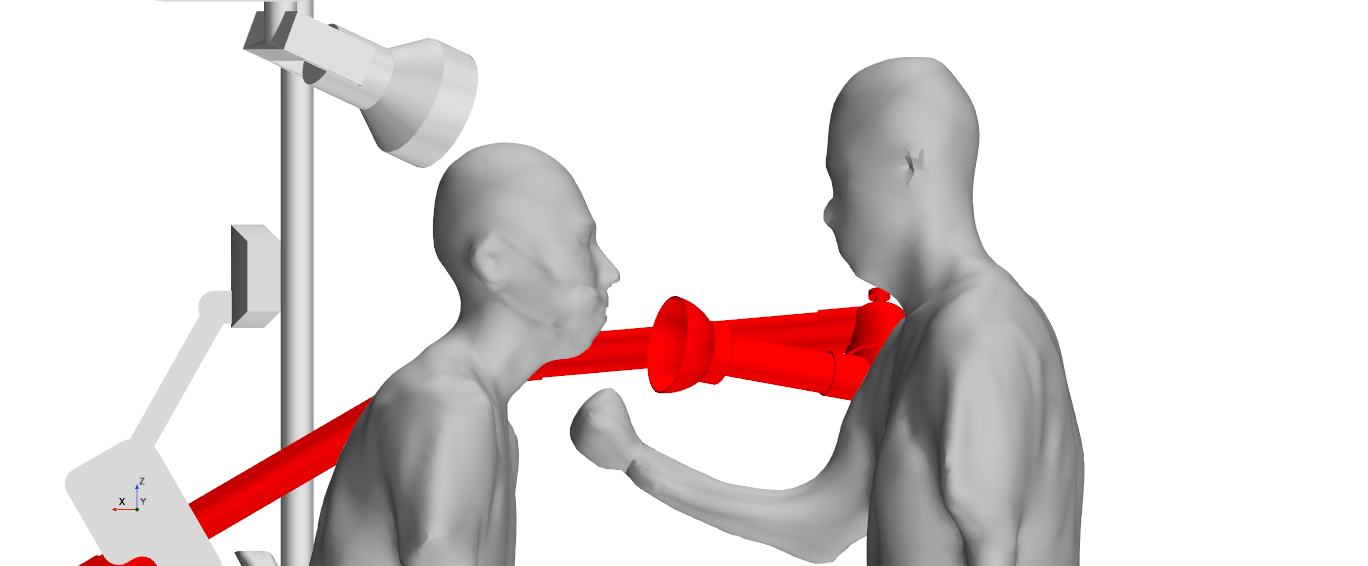


The suction port was placed approximately 15cm away from the patient's mouth


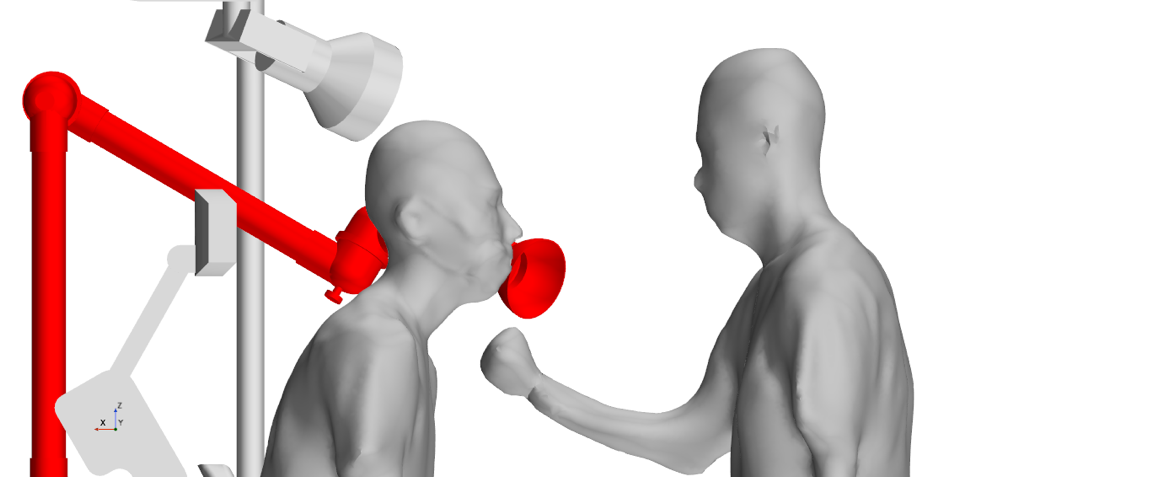


The suction port was placed near the patient's mouth


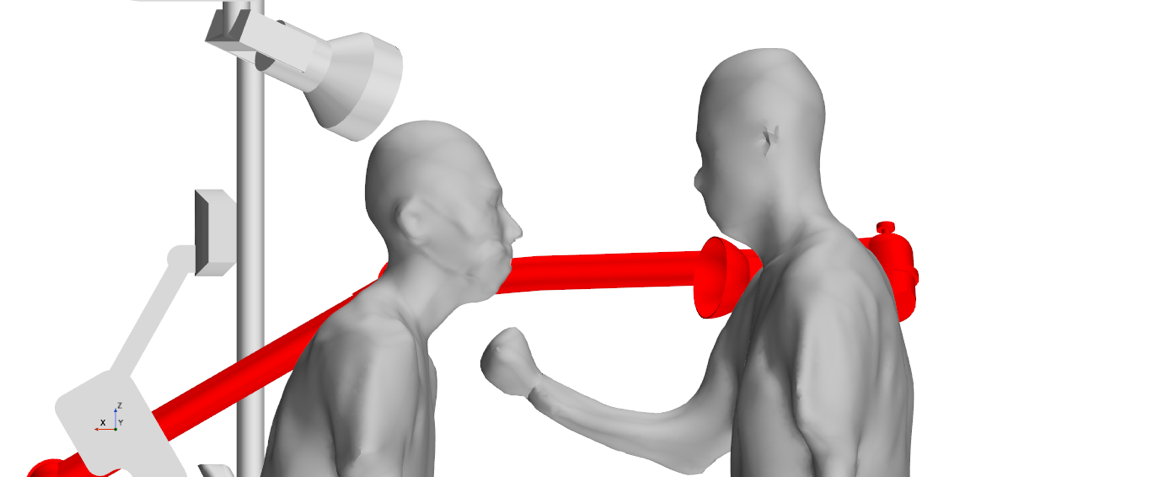


The suction port was placed approximately 35cm away from the patient's mouth

Figure S4. Three suction port arrangements modeled in this study.

| Without suction device | | | | | |
| --- | --- | --- | --- | --- | --- |
| RH50 | All particles | Floating | Adhere | Exhale | Evaporation |
| 10 µm | 5000 | 10 | 0 | 0 | 4990 |
|  | 100(%) | 0.2 | 0.0 | 0.0 | 99.8 |
| 80 µm | 5000 | 133 | 790 | 3 | 4074 |
|  | 100(%) | 2.7 | 15.8 | 0.1 | 81.4 |

Table S. Simulations with 33°C for the mouth temperature and 31°C for the body temperature

Setting conditions details:

Mouth temperature 33°C

Mouth humidity RH 70

Droplet temperature 33°C

Inspectors/inspectors 31°C

Room temperature 25°C

Room humidity RH50
